# Cluster Detection Tests in Spatial Epidemiology: A Global Indicator for Performance Assessment

**DOI:** 10.1371/journal.pone.0130594

**Published:** 2015-06-18

**Authors:** Aline Guttmann, Xinran Li, Fabien Feschet, Jean Gaudart, Jacques Demongeot, Jean-Yves Boire, Lemlih Ouchchane

**Affiliations:** 1 Department of Biostatistics, Clermont University Hospital, Clermont-Ferrand, France; 2 UMR CNRS UDA 6284 ISIT, Auvergne University, Clermont-Ferrand, France; 3 EA 7282 IGCNC, Auvergne University, Clermont-Ferrand, France; 4 UMR INSERM 912 SESSTIM, Aix-Marseille University, Marseille, France; 5 Faculty of Medicine of Grenoble FRE CNRS 3405 AGIM, J. Fourier University, La Tronche, France; INDEPTH Network, GHANA

## Abstract

In cluster detection of disease, the use of local cluster detection tests (CDTs) is current. These methods aim both at locating likely clusters and testing for their statistical significance. New or improved CDTs are regularly proposed to epidemiologists and must be subjected to performance assessment. Because location accuracy has to be considered, performance assessment goes beyond the raw estimation of type I or II errors. As no consensus exists for performance evaluations, heterogeneous methods are used, and therefore studies are rarely comparable. A global indicator of performance, which assesses both spatial accuracy and usual power, would facilitate the exploration of CDTs behaviour and help between-studies comparisons. The Tanimoto coefficient (TC) is a well-known measure of similarity that can assess location accuracy but only for one detected cluster. In a simulation study, performance is measured for many tests. From the TC, we here propose two statistics, the averaged TC and the cumulated TC, as indicators able to provide a global overview of CDTs performance for both usual power and location accuracy. We evidence the properties of these two indicators and the superiority of the cumulated TC to assess performance. We tested these indicators to conduct a systematic spatial assessment displayed through performance maps.

## Introduction

Assessing performance of local cluster detection tests (CDTs) is a complex but necessary task. For development of new statistical methods, simulation studies are obviously essential. In field investigation, they provide useful knowledge for interpretation of real data and decision making [1]. However, from a methodological point of view, there is still no commonly accepted protocol for simulation studies in spatial epidemiology. Evaluations are often incomplete as they are conducted only on a few clustering models which are defined by arbitrary settings that cannot reflect all the possible clustering configurations. Furthermore, performance, a critical aspect of which is the location accuracy, cannot be assessed just by usual power because it only measures the null hypothesis rejection. To address this issue, many different indicators of performance have been proposed.

Power and location accuracy are sometimes assessed separately using indicators purely dedicated to assess the location accuracy. These indicators are based on a 4-types spatial units (SUs) classification resulting from the confrontation between the detected cluster (positives or negatives SUs) and the simulated cluster (the *gold standard* leading to classification in true/false positives or negatives SUs). From this classification indicators such as sensitivity and positive predictive value are computed (for example see [2–6]). However, their mathematical definitions are heterogeneous. Some authors assess all clusters whether the null hypothesis is rejected or not [6], others only the detected clusters (*i.e.* with null rejection) [2, 5] and, finally, some authors also assess power by considering each analysis without null rejection as “no detected cluster” (*i.e.*, all SUs are false or true negatives) [4]. Other studies equally proposed concomitant assessment using conditional power, such as power-to-detect at least one spatial unit of the true cluster or power-to-detect exactly the true cluster (for example see [6–8]). As these indicators are based on very restrictive definitions, they only partially measure performance.

As only partial performance indicators are available, performance is usually assessed using a more or less large set of complementary indicators. Depending on the set of performance indicators used, interpretations and comparisons between studies might be difficult.

If the use of multiple indicators can provide very detailed information on CDTs behaviour, it also limits the number of clustering models that can be simulated. Indeed, a large number of clustering models results in a huge amount of information to treat and interpret, making it difficult to provide a comprehensible overview of performance. Even when clustering models are restricted by setting some parameters—such as relative risk and baseline incidence—in realistic ranges regarding the disease under study, global overview of performance is easier by measuring a single indicator. Such an indicator should obviously assess both power and location accuracy. However, what can be considered a sufficiently accurate test is quite ambiguous and depends on context. For example, one will need a far better accuracy for a secondary investigation than for a surveillance system. Thus, location accuracy should be measured with a quantitative indicator. In [9], we proposed the area under the curve of extended Power [10]. This indicator, while accounting for both usual Power and location accuracy, is complex.

This work is based on the coefficient developed by Tanimoto [11] (see also [12]). The Tanimoto coefficient (TC) is an easily comprehensible, fast computed indicator extensively used in image science [13–15] and biochemistry [16, 17]. The TC is a measure of similarity comparing two sample sets by using the ratio of the intersecting set to the union set. It is thus well suited to assess location accuracy for one detected cluster (*i.e.* the result of one test). To assess CDTs performance, we propose two statistics of the TC, both taking into account location accuracy and usual power in simulation studies. We conduct a systematic spatial assessment that, combined with these global measures, enables the building of performance maps.

The structure of this paper is as follows: in the Methods’ section, we describe each procedure of this simulation study following guidelines proposed by [18] when relevant. In the Results’ section, we present the performance of Kulldorff’s spatial scan statistic as measured by the proposed statistics. Finally, in the Discussion, we briefly compare these indicators with the area under the extended Power curve, discuss the behavior of these two statistics derived from the TC and argue the recommendation of the cumulated TC.

## Methods

### Clustering model

The study region is the Auvergne region (France), divided into n = 221 spatial units (SUs) equivalent to U.S. ZIP codes. For a realistic analysis, we used data archived in *CEMC* (birth defects registry for the Auvergne region) and *INSEE* (French Institute of Statistics and Economic Studies) databases. We collected two categories of data from 1999 to 2006: all birth defects and cardiovascular birth defects. For each SU, the number of live births (*i.e.*, the size of the at-risk population) was approximated by the number of birth declarations in the at-risk population. Global annual incidences of all birth defects and cardiovascular birth defects were estimated as 2.26% and 0.48% of births, respectively.

**Fig 1:**
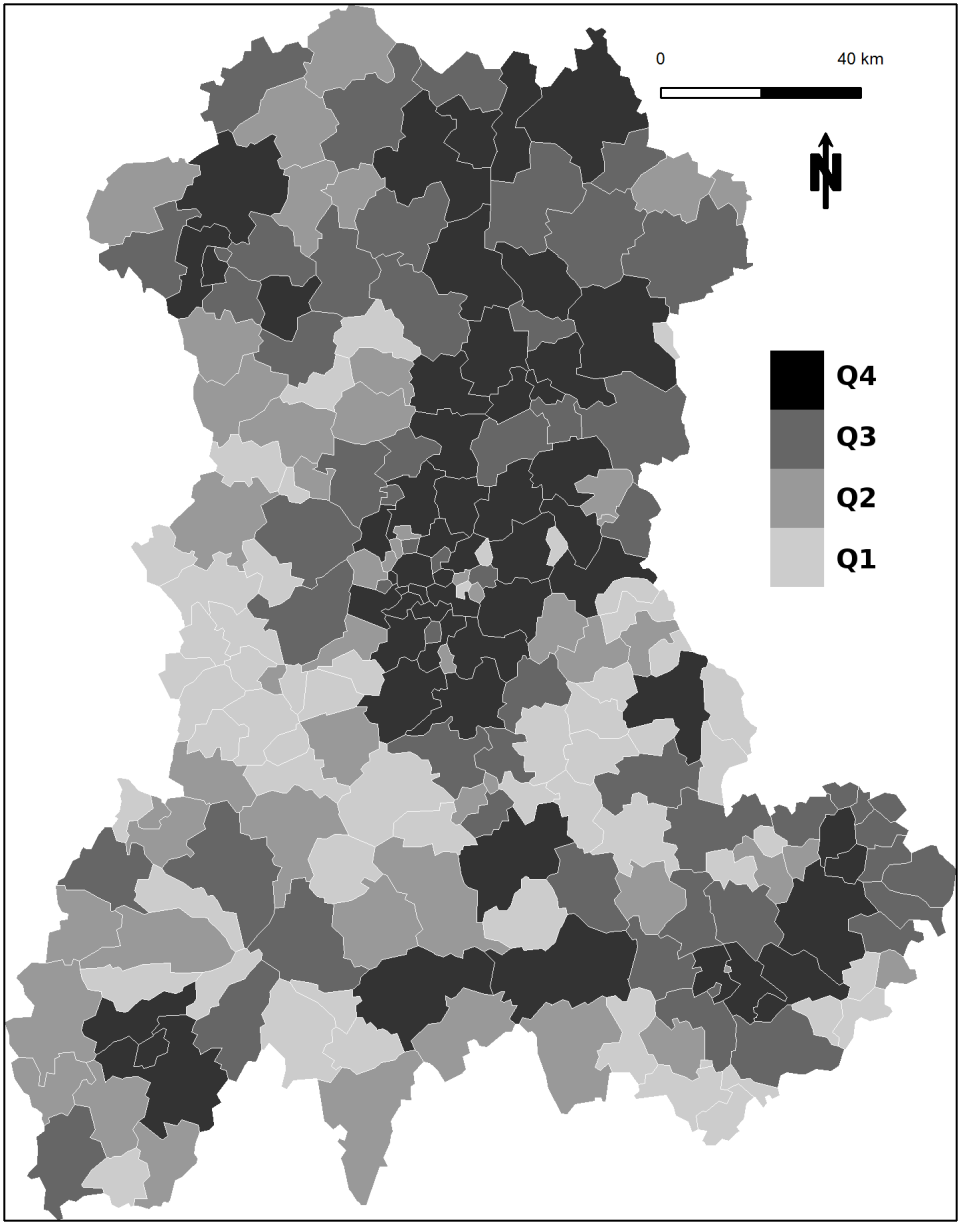
Size of the at-risk population for each SU in the Auvergne region, as defined by mean number of live births per year between 1999 and 2006 (source: INSEE). *Q*1:≤ 17; *Q*2:> 17 and ≤ 35; *Q*3:> 35 and ≤ 70; *Q*4:> 70.

We applied these two baseline risks (incidences) of birth defects to the same at-risk population, which size was approximated by mean annual number of live births. (The distribution of the at-risk population is shown in Fig 1.) For each baseline incidence (*I* = 2.26% of births or *I* = 0.48%), we defined two cluster collections by applying two relative risks (3 and 6) to the same pattern of location and cluster size. The relative risks were chosen in order to observe all the range of performance. Each cluster collection contains 221 clusters of four SUs (one central SU and its three nearest neighbors in euclidean distances) successively centered on each SU of the region.

### Datasets

We generated 1000 datasets for each combination of baseline risk, relative risk and cluster location, i.e. a total of 884 000 datasets.

Each dataset is a table of 221 rows and 5 columns. The rows contain the coordinates (longitude and latitude) of a SU, the observed number of cases, the size of the at-risk population (i.e., the number of live births) and the expected number of cases in the specified SU assuming an inhomogeneous Poisson process for the cases distribution. The expected number of cases is the product of the global incidence (*I* = 2.26% or *I* = 0.48%) and the size of the at-risk population in the SU. The observed case numbers are assumed as independent Poisson variables such that

$$\left\{ \begin{array}{cc} H_{0}:N_{i}∼\text{Pois}(\varepsilon_{i}),i=1,...,n & \\ H_{1}:N_{i}∼\text{Pois}(\pi_{i}),\pi_{i}=\varepsilon_{i}[1+𝕀(\theta -1)],i=1,...,n \end{array}\right.$$

where *N*_*i*_ is the observed number of cases, *ɛ*_*i*_ denotes the expected number of cases in the ith SU under the null hypothesis of risk homogeneity (*H*_0_) and *π*_*i*_ the expected number of cases in the ith SU under the alternative hypothesis of one simulated cluster (*H*_1_), *θ* is the relative risk, and 𝕀 is a binary indicator set to 1 if the ith SU is within the simulated cluster, and 0 otherwise.

We used the R function “rpois” [19] with the default Mersenne-Twister pseudo-random number generator developed by Matsumoto [20]. For reproducibility purpose, all datasets were archived.

### Statistical programming

Statistical programming was done with R 3.0.2 64 bits using the “SpatialEpi” library [21] and the “kulldorff” function to perform the analysis.

In order to optimize computational time, we used parallel programming through the function “foreach” of package “Foreach” [22] with the parallel backend provided by the package “DoSNOW” [23]. Computation were done on a Dell T7600 (processor Intel(R) Xeon CPU ES-2620 2 GHz and 32 Go RAM).

### Kulldorff’s spatial scan statistic

In this study, we selected Kulldorff’s spatial scan statistic [24, 25] as a well-known and widely used CDT which performance has been studied by many authors [6, 26–28]. The spatial scan statistic detects the most likely cluster on locally observed statistics of likelihood ratio tests. The scan statistic considers all possible zones *z* defined by two parameters: a center that is successively placed on the centroid of each SU, and a radius varying between 0 and a predefined maximum. The true geography being delineated by administrative tracts, each zone *z*, defined by all SUs which centroids lie within the circle, is irregularly shaped. Let *N*_*z*_ and *n*_*z*_ be the size of the at-risk population and the number of cases counted in zone *z*, respectively (over the whole region, these quantities are the total population size *N* and the total number of cases *n*). The probabilities that a case lies inside and outside zone *z* are defined by $p_{z}=\frac{n_{z}}{N_{z}}$ and $q_{z}=\frac{\left(n-n_{z}\right)}{\left(N-N_{z}\right)}$, respectively. Given the null hypothesis of risk homogeneity *H*_0_: *p*_*z*_ = *q*_*z*_, versus the alternative *H*_1_: *p*_*z*_ = *q*_*z*_ and assuming a Poisson distribution of cases, the likelihood ratio statistics are defined as proportional to ${\left(\frac{n_{z}}{\lambda N_{z}}\right)}^{n_{z}}{\left(\frac{n-n_{z}}{\lambda \left(N-N_{z}\right)}\right)}^{n-n_{z}}𝕀\left[n_{z}>\lambda N_{z}\right]$, where *λ* is the annual incidence *I* (here equal to 2.26% or 0.48%) and the indicator function 𝕀 equals 1 when the number of observed cases in zone *z* exceeds the expected number under *H*_0_ of risk homogeneity, and 0 otherwise. The circle yielding the highest likelihood ratio is identified as the most likely cluster. The p-value is obtained by Monte Carlo inference.

Over the 884 000 simulated datasets, each test was performed with a maximum size of zone *z* set to 50% of the total at-risk population, a number of 999 Monte Carlo samples for significance measures, and alpha risk set to 5%.

### Measure of performance

For each simulation, in order to compute the performance measures, we stored the identifiers of the SUs in the most likely cluster and the corresponding estimated *p-value*. As Monte Carlo hypothesis testing is based on simulations, there is no guarantee that *p-values* would be exactly the same for successive analyses of the same datasets. For reproducibility purpose, the aforementioned results were thus archived along with the original datasets.

#### Tanimoto coefficient

The TC was computed for each analysed dataset. This coefficient measures the similarity between the simulated cluster and the detected cluster. The superimposing of these two clusters leads to the definition of four types of SUs. The SUs both within the simulated and the detected cluster are true positives (*TP*), the SUs only within the detected cluster are false positives (*FP*), the SUs only within the simulated cluster are false negatives (*FN*) and, finally, the SUs within neither cluster are true negatives (*TN*). When no cluster was detected, i.e. *p-value* higher than 0.05, all 221 SUs were considered negatives and the analysis resulted in *TP* = 0, *FP* = 0, *TN* = 217, *FN* = 4.

The *TC*, computed for each analyzed dataset, is such that $TC=\frac{TP}{TP+FP+FN}$. For each simulated cluster, 1000 datasets were analyzed, and thus 1000 *TC* were computed.

We defined two statistics of *TC*, both ranging between 0 and 1, in order to obtain two performance measures for each simulated cluster (with a total of 884 clusters).

#### Averaged Tanimoto coefficient

This first summary statistic of *TC*, referred to as *TC*_*a*_ is the arithmetic mean of all *TC* over the *m* simulated datasets. It is defined as

$$TC_{a}=\frac{1}{m}\times \sum_{i=1}^{m}\frac{TP_{i}}{TP_{i}+FP_{i}+FN_{i}}.$$

#### Cumulated Tanimoto coefficient

The second summary statistic, the *TC*_*c*_, is the cumulated *TC* over the *m* simulated datasets, and is defined as

$$TC_{c}=\frac{\sum_{i=1}^{m}TP_{i}}{\sum_{i=1}^{m}TP_{i}+FP_{i}+FN_{i}}.$$

### Performance mapping

Following a previous study [9], global performance is visualised over the entire region using maps representing the *TC_a_* and *TC_c_* for each collection of clusters.

Each of these measures corresponds to one measure of a cluster and thus is associated with four SUs. In order to obtain a global overview on a single map, we assigned the performance measure for one cluster to its central SU. We thus affected a single measure of performance to each SU of the map. As we defined four cluster collections for four risks combinations (incidence and relative risks), we produced four performance maps for each indicator.

## Results

### Performance maps

**Fig 2:**
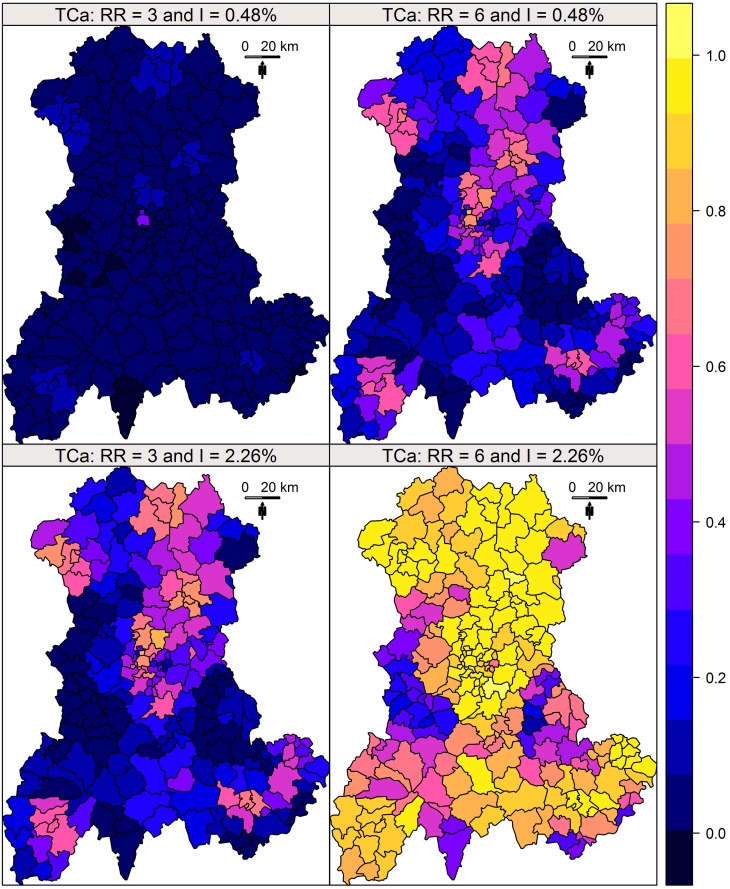
*TC_a_* of Kulldorff’s spatial scan. *TC_a_* measured for four combinations of two relative risks (RR) and two annual incidences of birth defects: low RR = 3 and high RR = 6; low incidence = 0.48% births per year and high incidence = 2.26% births per year.

**Fig 3:**
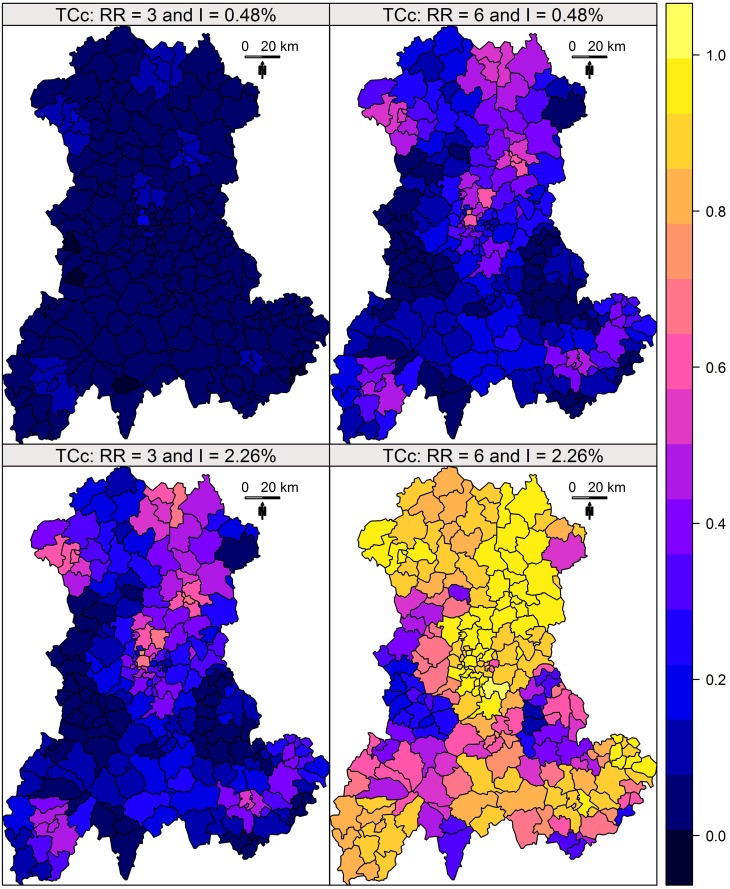
*TC_c_* of Kulldorff’s spatial scan. *TC_c_* measured for four combinations of two relative risks (RR) and two annual incidences of birth defects: low RR = 3 and high RR = 6; low incidence = 0.48% births per year and high incidence = 2.26% births per year.

**Fig 4:**
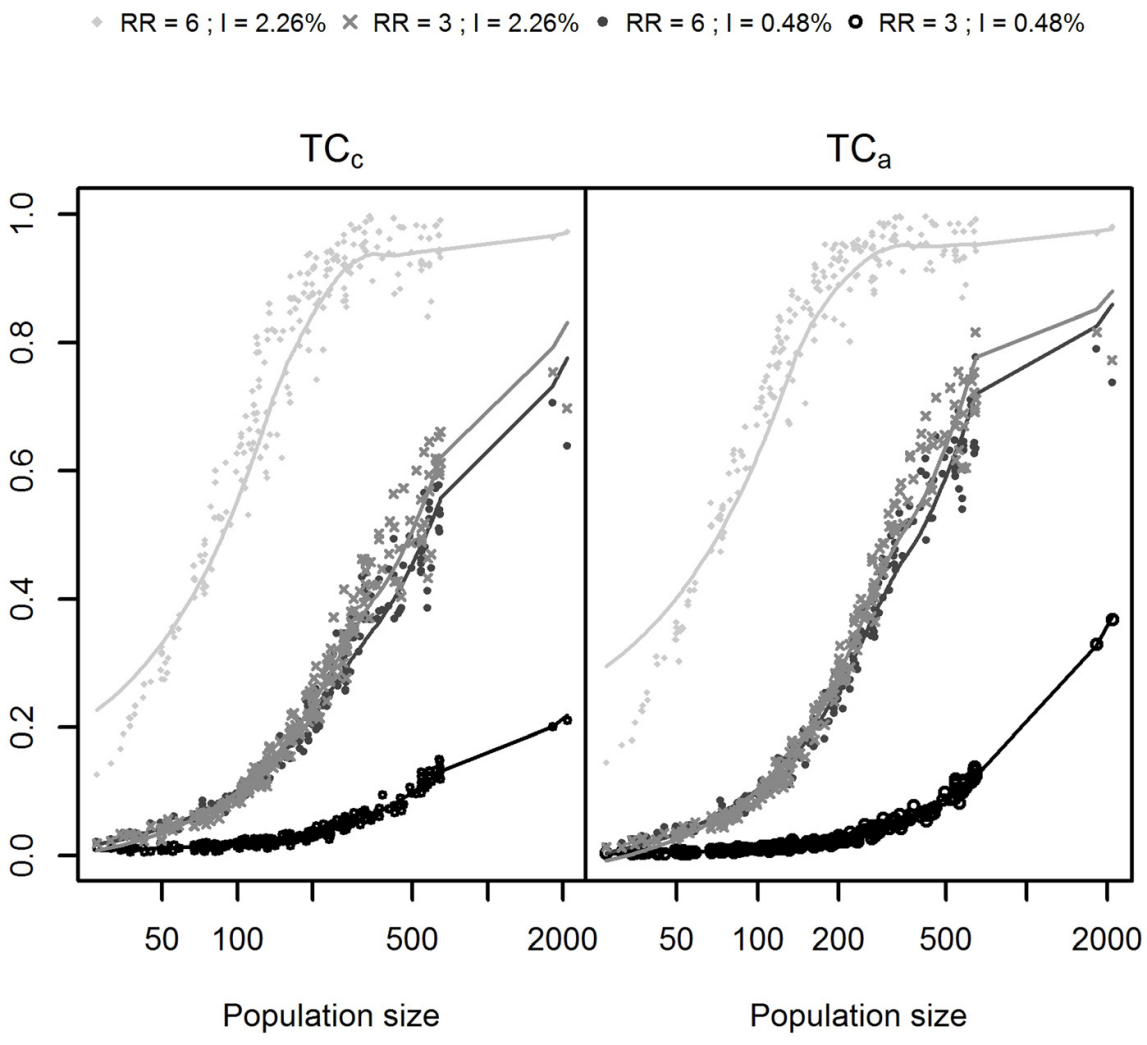
Performance indicators and size of at-risk population. Indicators are measured for four combinations of two relative risks (RR) and two annual incidences of birth defects: low RR = 3 and high RR = 6; low incidence = 0.48% births per year and high incidence = 2.26% births per year.

**Fig 5:**
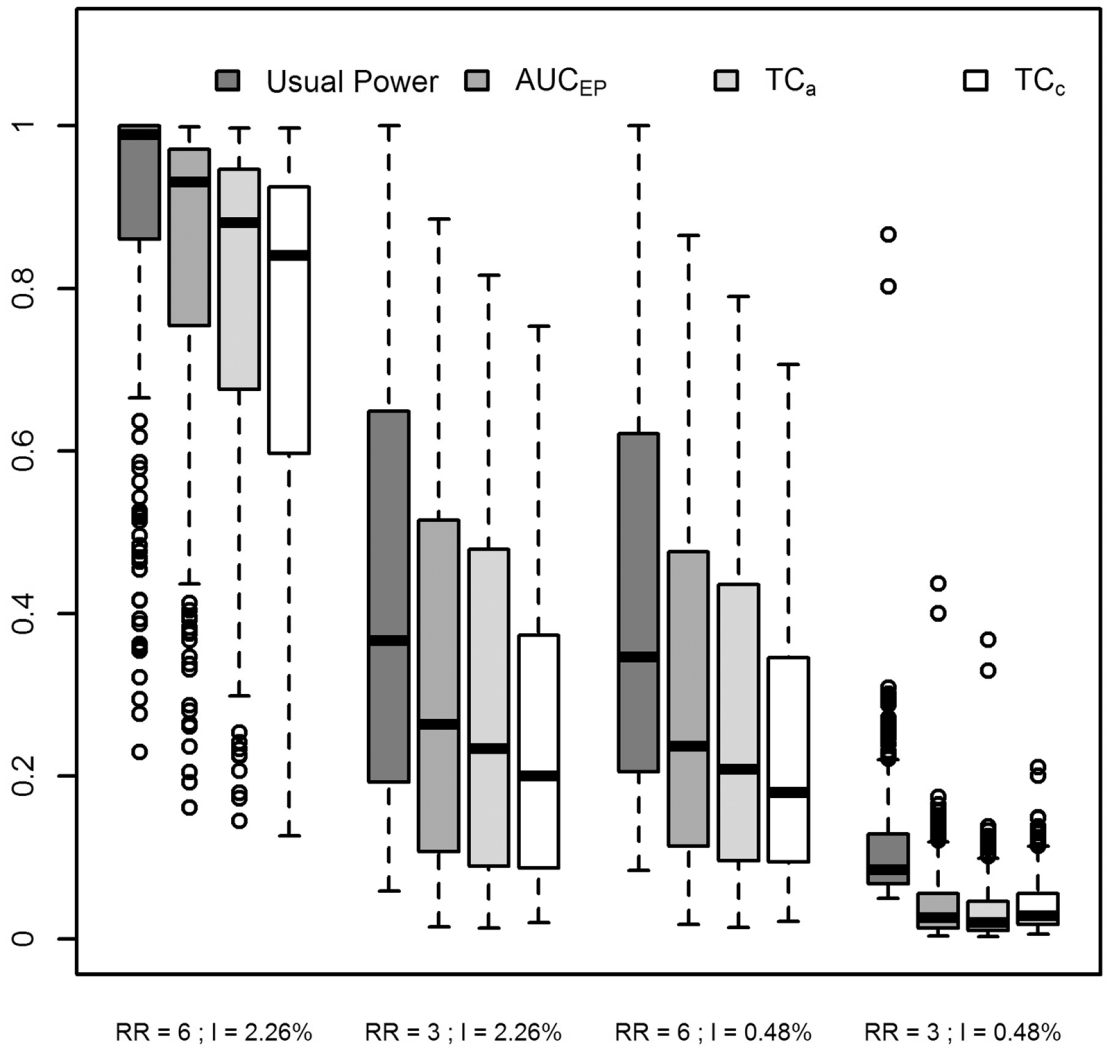
Summary statistics of usual Power, *AUC_EP_*, *TC_a_* and *TC_c_*. Results for four combinations of two relative risks (RR) and two annual incidences of birth defects: low RR = 3 and high RR = 6; low incidence = 0.48% births per year and high incidence = 2.26% births per year.

The results of this simulation study are shown in Figs 2 and 3. Whatever the indicator, the performance was heterogeneously distributed, in close relationship with the size of the at-risk population (Fig 4). The distributions of the *TC*_*a*_ and *TC*_*c*_ for each risks level are described in Fig 5.

### Averaged Tanimoto coefficient versus cumulated Tanimoto coefficient

**Fig 6:**
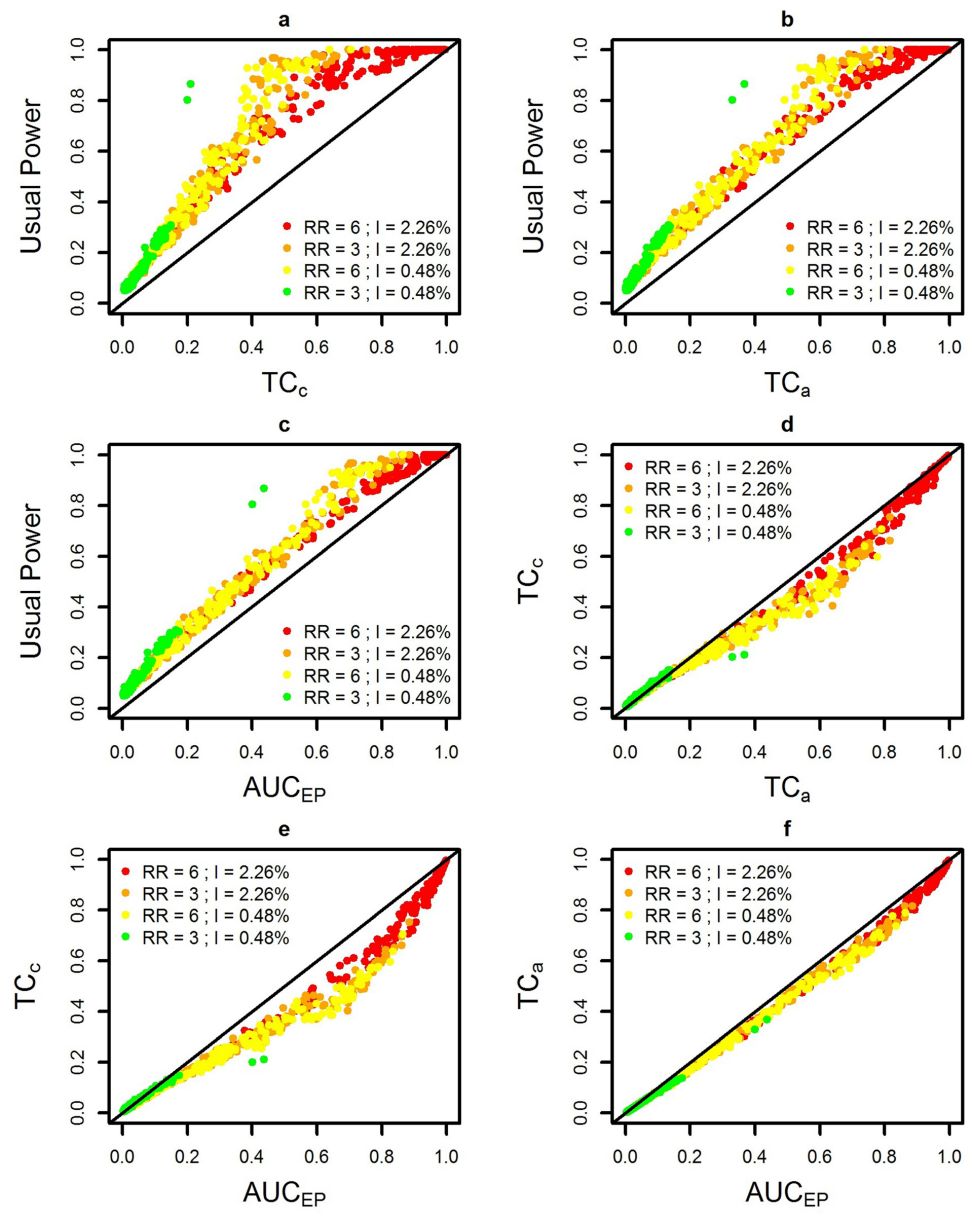
Performance measures for four combinations of two relative risks (RR) and two annual incidences of birth defects: low RR = 3 and high RR = 6; low incidence = 0.48% births per year and high incidence = 2.26% per year births. (a) Usual Power and *TC_c_*, (b) Usual Power and *TC_a_*, (c) Usual Power and *AUC_EP_*, (d) *TC_c_* and *TC_a_*, (e) *TC_c_* and *AUC_EP_*, (f) *TC_a_* and *AUC_EP_*

The *TC*_*c*_ was generally lower than the *TC*_*a*_, that is, the test performance is judged as less by the *TC*_*c*_ (see Fig 6d). For *RR* = 6 with *I* = 2.26%, *RR* = 3 with *I* = 2.26% and *RR* = 6 with *I* = 0.48%, the *TC*_*c*_ was lower than the *TC*_*a*_ in 100%, 74.7% and 75.6% of simulations, respectively. On the contrary, for *RR* = 3 with *I* = 0.48%, i.e. the lowest risks level, the *TC*_*c*_ was higher than *TC*_*a*_ in 97.3% of simulations.

Fig 6a and 6b show *TC*_*c*_ and *TC*_*a*_ compared with the usual Power. Usual Power was always higher than both the *TC*_*c*_ and *TC*_*a*_, as was expected. Indeed, each detected cluster (most likely cluster with significant *p-value*) always contributes for 1 in the usual Power, but it contributes for 1 in the *TC*_*c*_ or *TC*_*a*_ only if the detected cluster is exactly the same as the simulated cluster, and less than 1 otherwise.

With both *TC*_*c*_ and *TC*_*a*_, the spatial scan showed comparable performance on the two intermediate levels of risks (*RR* = 3 with *I* = 2.26% and *RR* = 6 with *I* = 0.48%) and a poor performance on the lowest level of risks (*RR* = 3 with *I* = 0.48%). The *TC*_*c*_ showed more variability than *TC*_*a*_ when the spatial scan was the most efficient in terms of usual power (see Fig 6a and 6b).

## Discussion

Both indicators enable the construction of performance maps, providing a global overview of Kulldorff’s spatial scan performance.

**Fig 7:**
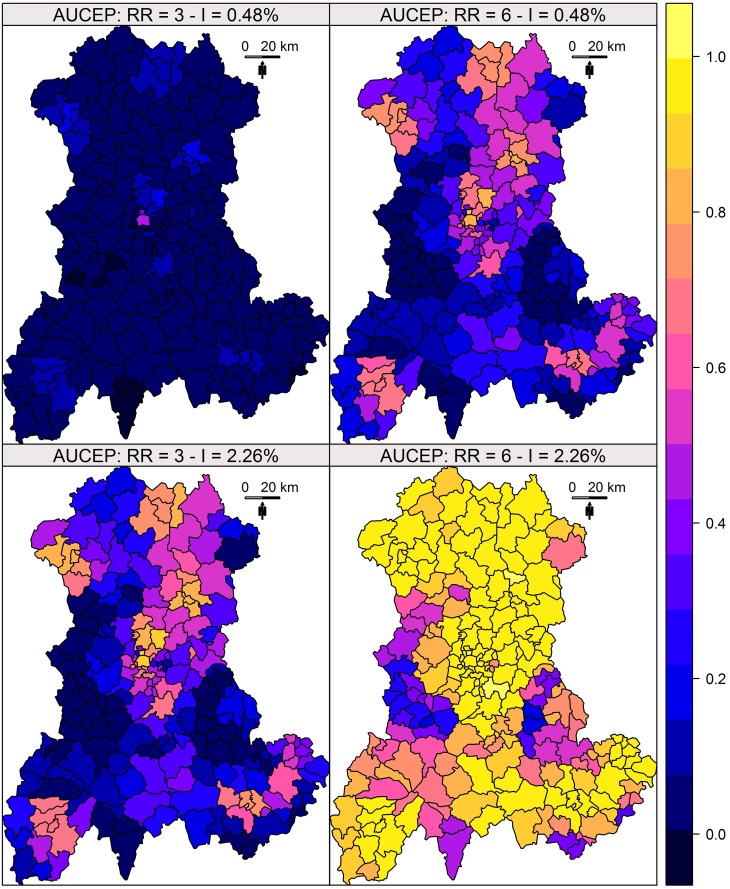
*AUC_EP_* of Kulldorff’s spatial scan. *AUC_EP_* was measured for four combinations of two relative risks (RR) and two annual incidences of birth defects: low RR = 3 and high RR = 6; low incidence = 0.48% births per year and high incidence = 2.26% births per year.

In a previous study [9], we used the area under the curve of extended Power (*AUC_EP_*), whose concept and construction are described in Takahashi *et al.* [10]. Compared to this previous study (Fig 7), the results of the current study are very similar, especially considering *TC_a_* (see Fig 6b, 6c and 6f). However, both *TC_a_* and *TC_c_* indicate a lower performance of the test (see Fig 6e and 6f).

The test performance was judged as less by the *TC_c_* than either the *TC_a_* or the *AUC_EP_* (see Fig 6d and 6e), except for the lowest risks level where this order relation is reversed.

Ideally, we would already dispose of a gold standard capable of measuring the true performance of the test. As this is not the case, we cannot compare the observed *TC_a_* and *TC_c_* to determine the one closer to the true performance. Thus, simply observing a lower or higher value of *TC_a_* compared to *TC_c_* cannot be used as an objective argument in favour or disfavour of one indicator. However, the systematic nature of the relationship between *TC_a_* and *TC_c_* must be explained, as its reasons are the only objective arguments on which to base a decision to recommend one over the other.

In order to understand this behaviour, we considered the functions *f*(*s*) and *g*(*s*) representing the computation at simulation *s* of respectively *TC_a_* and *TC_c_*. The simulations are sorted as follows: *(i)*, the *s* = 1 to *q* simulations resulting in cluster detection, *i.e.* with *p-value* < 0.05, are sorted by increasing number of *FP*;*(ii)*, the remaining simulations (*s* = *q* + 1 to *m*′) are sorted without particular order as they result in the exact same assessment of performance (*TP* = 0, *FP* = 0, *TN* = 217, *FN* = 4).

**Fig 8:**
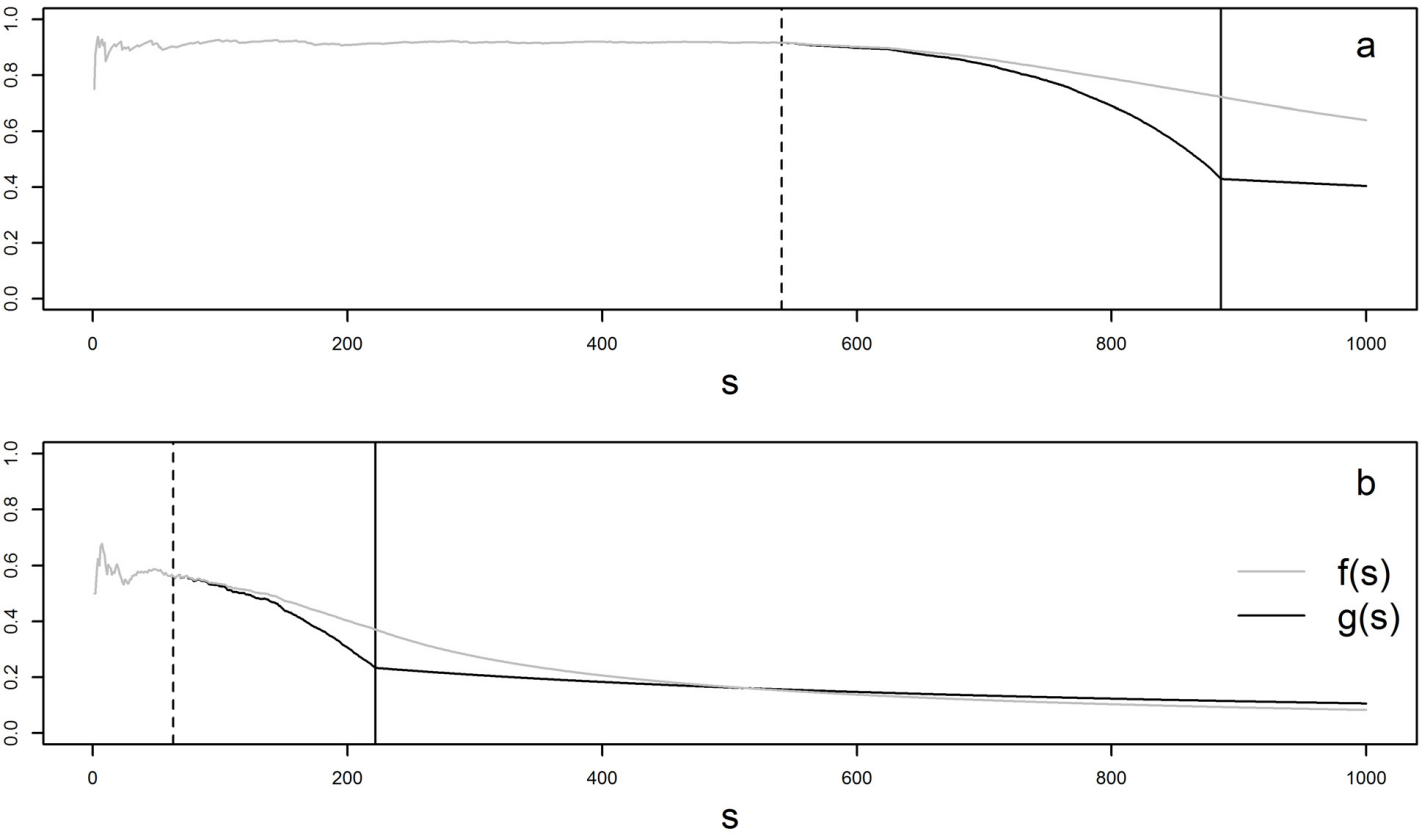
Values of *f(s)* and *g(s)* for simulation *s = 1: 1000*. The simulations displayed before the vertical plain line lead to null rejection (*p*—*value* < 0.05). They are sorted by increasing number of FP SUs. The dotted line represent the last simulation resulting in a detected cluster without FP SUs. The functions *f*(*s*) and *g*(*s*) represent respectively the computation of *TC_a_* and *TC_c_* over the *m*′ simulations. (a) simulated cluster with the maximum value of *TC_a_*—*TC_c_* and (b) simulated cluster with the minimum value of *TC_a_*—*TC_c_*.

Fig 8 shows two examples of curves defined by *f*(*s*) and *g*(*s*). Fig 8a corresponds to the simulated cluster with the maximum value of *TC_a_*—*TC_c_* and Fig 8b corresponds to the one with the minimum value of *TC_a_*—*TC_c_*.

At the simulation q, *f*(*q*) is equal to

$$\begin{array}{ccc} f(q) & = & \frac{\sum_{s=1}^{q}\frac{TP_{s}}{TP_{s}+FN_{s}+FP_{s}}}{q}\\ & = & \frac{\sum_{s=1}^{q}\frac{TP_{s}}{D+FP_{s}}}{q}\\ & = & \sum_{s=1}^{q}\frac{TP_{s}}{qD+qFP_{s}}, \end{array}$$

where *D* is the number of SUs in the simulated cluster (by definition D is constant in our simulations). The value of *g*(*s*) at the simulation q is equal to

$$g\left(q\right)=\frac{\sum_{s=1}^{q}TP_{s}}{\sum_{s=1}^{q}TP_{s}+FN_{s}+FP_{s}}=\frac{\sum_{s=1}^{q}TP_{s}}{qD+\sum_{s=1}^{q}FP_{s}}.$$

These two equations, easily explain the first part of the curves shown in Fig 8. Indeed, when a detected cluster does not contain FP (up to the dotted line), these equations are strictly equivalent and the two curves are superposed.

From the results of the 884 simulations conducted in this study, we first note that, *f*(*q*) was always strictly greater than the corresponding *g*(*q*). This relationship can be explained by partitioning the q simulations in three disjoint sets: *S*_0_ = {*s*|*TP_s_* = 0}, *S*_1_ = {*s*|*FP_s_* = 0} and *S*_2_ = {*s*|*TP_s_* ≠ 0 *and* *FP_s_* ≠ 0}. (In the first q simulations, a cluster is always detected and thus true and false positives can never be both null.) We can then write

$$f\left(s\right)=\sum_{S_{1}}^{}\frac{TP_{s}}{qD}+\sum_{S_{2}}^{}\frac{TP_{s}}{qD+qFP_{s}}$$ (1)

and

$$g\left(s\right)=\frac{\sum_{S_{1}}^{}TP_{s}+\sum_{S_{2}}^{}TP_{s}}{qD+\sum_{S_{0}}^{}FP_{s}+\sum_{S_{2}}^{}FP_{s}},$$

or equivalently

$$\begin{array}{ccc} g(s=q) & = & \frac{\sum_{S_{1}}^{}TP_{s}}{qD+\sum_{S_{0}}^{}FP_{s}+\sum_{S_{2}}^{}FP_{s}}\\ & \; & +\frac{\sum_{S_{2}}^{}TP_{s}}{qD+\sum_{S_{0}}^{}FP_{s}+\sum_{S_{2}}^{}FP_{s}} \end{array}$$ (2)

**Fig 9:**
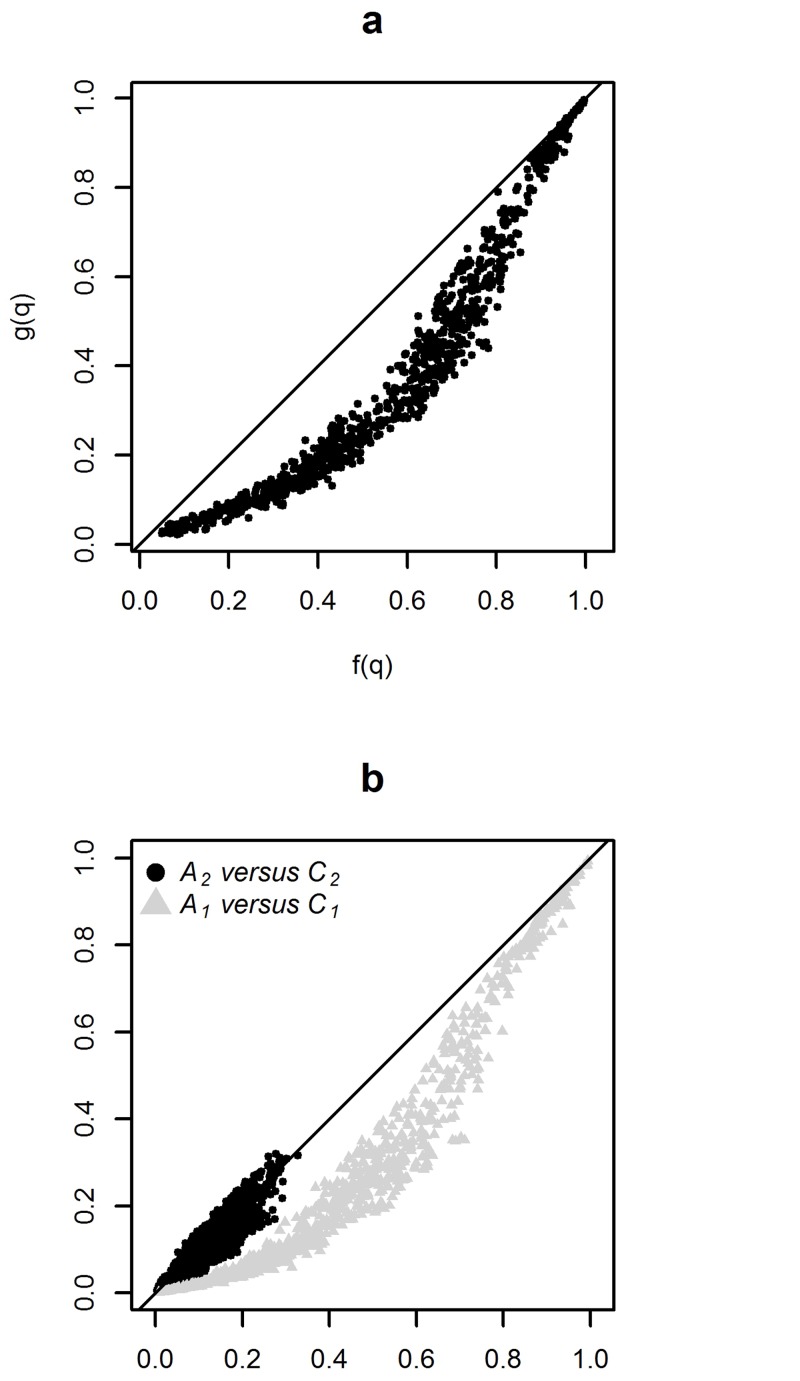
Relationship between *f(s)* and *g(s)* at simulation *s = q*. (a) *f*(*q*) versus *g*(*q*) and (b) Contribution of each term of the sums *f*(*q*) = *A*_1_ + *A*_2_(in ordinate) and *g*(*q*) = *C*_1_ + *C*_2_ (in abscissa). With $A_{1}=\sum_{S_{1}}^{}\frac{TP_{s}}{qD}$, $C_{1}=\frac{\sum_{S_{1}}^{}TP_{s}}{qD+\sum_{S_{0}}^{}FP_{s}+\sum_{S_{2}}^{}FP_{s}}$, $A_{2}=\sum_{S_{2}}^{}\frac{TP_{s}}{qD+qFP_{s}}$ and $C_{2}=\frac{\sum_{S_{2}}^{}TP_{s}}{qD+\sum_{S_{0}}^{}FP_{s}+\sum_{S_{2}}^{}FP_{s}}$.

It is then easy to show graphical proof that the first terms of the sums in Eqs (1) and (2), referred to as *A1* and *C1* respectively in Fig 9, determine the order relation between *f*(*q*) and *g*(*q*). (The second terms of the sums in Eqs (1) and (2) are referred to as *A2* and *C2* respectively.) In fact, simulations where there is no *TP* do not impact *f*(*q*) but decrease *g*(*q*) all the more so due to the *FP*. Also, *g*(*q*) decreases more strongly than *f*(*q*) with higher number of *FP*. As the mean number of *FP* (for all 884 simulated clusters) is 11.07 (median 4) when there is no *TP* and 6.5 (median 0 and third quartile 3) when there is at least one *TP*, the order relation (*f*(*q*) > *g*(*q*)) is explained.

Our second observation is that *TC_c_* (*i.e.* *g*(*s* = *m*′)) is less than *TC_a_* (*i.e.*, *f*(*s* = *m*′)), except for the lowest risks level. To explain this, let now consider any simulation s, where *s* > *q*. As no cluster is detected, there are neither false nor true positives and the quantities $M=\sum_{s=1}^{q}\frac{TP_{s}}{D+FP_{s}}$, $A=\sum_{s=1}^{q}TP_{s}$ and $B=\sum_{s=1}^{q}FP_{s}$ are equal to $\sum_{s=1}^{m^{\prime }}\frac{TP_{s}}{D+FP_{s}}$, $\sum_{s=1}^{m^{\prime }}TP_{s}$ and $\sum_{s=1}^{m^{\prime }}FP_{s}$, respectively. Thus, we can write

$$f\left(s>q\right)=\frac{M}{s}$$

and

$$g\left(s>q\right)=\frac{A}{B+qD+(s-q)D}=\frac{A}{B+sD}.$$

The asymptotic behavior of the ratio of *f*(*s*) to *g*(*s*), is then

$$\begin{array}{ccc} \lim_{s\to \infty }\frac{f(s)}{g(s)} & = & \lim_{s\to \infty }\frac{M}{s}\times \frac{B+sD}{A}\\ & = & \lim_{s\to \infty }\{\frac{M}{A}\times (\frac{B}{s}+D)\}\\ & = & \frac{MD}{A}, \end{array}$$

as $\frac{B}{s}$ tends to 0. As *M* can only be less than or equal to $\frac{A}{D}$, then $\lim_{s\to \infty }\frac{f(s)}{g(s)}$ is less than or equal to 1. When there is at least one *FP* in the first *q* simulations, then $\frac{A}{D}$ is strictly greater than M and $\lim_{s\to \infty }\frac{f(s)}{g(s)}$ is strictly less than 1. That is, *TC_a_* is less impacted by simulations where no cluster are detected (*p-value* ≥ 0.05), explaining the higher final values of *TC_c_* compared to *TC_a_* for the lowest risk levels where usual Power is of 11.7% on average.

The absence of *TP*, or a high number of *FP* when a cluster is detected, reflects a poor performance and should negatively impact the indicators. As the contributions of these simulations are much stronger in *TC_c_* than in *TC_a_*, *TC_c_* better distinguishes low accuracy in cluster location. Furthermore, even if *TC_a_* is generally lower than *TC_c_* when the usual power is very low, the range of values reflects unambiguously low performance. Finally, *TC_c_* can be directly interpreted like the original Tanimoto coefficient, *i.e.* a measure of similarity comparing two sample sets by using the ratio of the intersecting set to the union set where the two sets are the stacked results of the simulations. For these reasons, we recommend the use of *TC_c_* to assess CDTs performance.

This type of study is generally undertaken for a purpose of research or to prepare for the deployment of a health monitoring system. In this context, long computational time can be tolerated, as there is no need to repeat the study. Nevertheless, a systematic spatial assessment of a CDT performance in detecting a type of cluster (fixed shape, size and epidemiological factors) is bound to be time-costing. In this study, the simulation and analysis of the 221 000 datasets necessary for the construction of one map required about 43 hours of computation. Most of this time was taken by the analysis of the datasets by the CDT, however. Once obtained the characteristics of the detected clusters, computation of the performance indicators and construction of the maps were relatively short (less than half an hour). Thus, using the cumulated Tanimoto coefficient would not substantially extend computational time of simulations studies conducted with a language faster than R, and analyses of results from previous simulation studies should be fast enough.

Many statistical methods are available to analyse spatial and temporal data. Quality of monitoring system or epidemiological research does not depend *per se* on the performance of these methods, but on how well their performance is known. Indeed, such knowledge is essential to chose appropriate methods and to interpret results. Every new or improved CDT is proposed along with an assessment of its performance. However, there is neither consensus nor commonly used methodology for performance evaluation. Then studies are rarely comparable and each new performance assessment must repeat assessment of the same reference CDTs in order to dispose of interpretable results. A sensible gain could be obtained by homogenisation of assessment methods. Furthermore, the use of a global performance indicator would allow for a great number of simulations, while still being able to communicate findings in a concise, comprehensible manner with a clear interpretation. We here propose a global performance indicator taking into account both usual Power and location accuracy and easy to compute and interpret. Finally, the cumulated Tanimoto coefficient can be used as is for assessment of performance on temporal data, and can be easily adapted to spatio-temporal data.
